# Supplementary material for: Calprotectin and Imbalances between Acute-Phase Mediators Are Associated with Critical Illness in COVID-19
Source: Int J Mol Sci. 2022 Apr 28;23(9):4894. doi: 10.3390/ijms23094894 (PMC9099708; doi:10.3390/ijms23094894)
Supplement: Supplementary file 1 [file ijms-23-04894-s001.zip › ijms-1656034-SI.pdf]

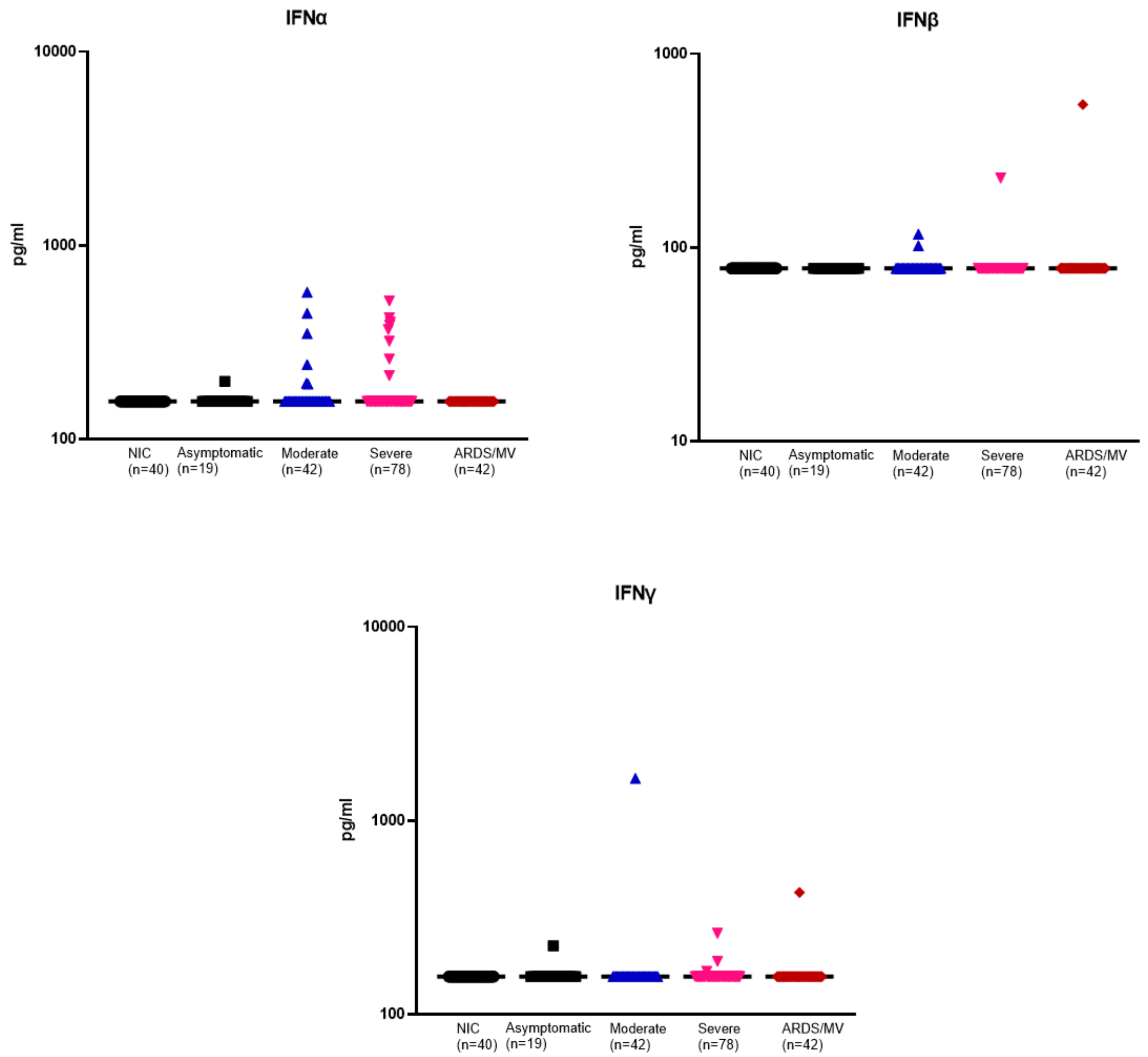

Figure S1. Concentrations of interferons. Dot plots with horizontal lines indicating the median of each group. The numbers of subjects evaluated are listed in the parentheses. Double arrowhead lines indicate comparisons between groups. Only statistically significant differences are indicated as follows: *p <0.05; ** p <0.01; ***p< 0.001; ****p< 0.0001. Abbreviations: ARDS= acute respiratory distress syndrome; IFN= interferon; MV= mechanical ventilation; n=number of patients; NIC= non-infected comparators
